# Immigrant-native differences in caries-related knowledge, attitude, and oral health behaviors: a cross-sectional study in Taiwan

**DOI:** 10.1186/1472-6831-14-3

**Published:** 2014-01-14

**Authors:** Chih-Chang Chen, Shang-Jyh Chiou, Chun-Chan Ting, Ying-Chun Lin, Chih-Cheng Hsu, Fu-Li Chen, Chien-Hung Lee, Ted Chen, Chin-Shun Chang, Ya-Ying Lin, Hsiao-Ling Huang

**Affiliations:** 1 Department of Oral Hygiene, College of Dental Medicine, Kaohsiung Medical University, 100 Shih-Chuan 1st Road, Kaohsiung 80708, Taiwan; 2 Institute of Occupational Safety and Health, Council of Labor Affairs, Executive Yuan, New Taipei City, Taiwan; 3 Department of Health Care Management, College of Healthcare Administration and Management, National Taipei University of Nursing and Health Sciences, Taipei, Taiwan; 4 School of Dentistry, College of Dental Medicine, Kaohsiung Medical University, Kaohsiung, Taiwan; 5 Institute of Population Health Sciences, National Health Research Institutes, Miaoli County 350, Taiwan; 6 Department of Public Health, College of Medicine, Fu Jen Catholic University, New Taipei City, Taiwan; 7 Department of Public Health, College of Health Science, Kaohsiung Medical University, Kaohsiung, Taiwan; 8 Department of Global Community Health and Behavioral Sciences, School of Public Health and Tropical Medicine, Tulane University, 70112 New Orleans, LA, USA; 9 Global Center of Excellence for Oral Health Research and Development, Kaohsiung Medical University, Kaohsiung, Taiwan; 10 Department of Nursing, Shu-Zen College of Medicine and Management, Kaohsiung, Taiwan

**Keywords:** Attitudes, Behavior, Dental caries, Immigrants, Health care

## Abstract

**Background:**

With the growing number of transnational marriages in Taiwan, oral health disparities have become a public health issue. This study assessed immigrant-native differences in oral health behaviors of urban mothers and their children.

**Methods:**

We used the baseline data of an oral health promotion program to examine the immigrant-native differences in caries-related knowledge, attitude, and oral health behaviors. A cross-sectional study was conducted to collect data from mothers in urban area, Taiwan. A total of 150 immigrant and 440 native mothers completed the self-report questionnaires. Logistic regression models analyzed the racial differences in oral health behaviors.

**Results:**

Approximately 37% of immigrant mothers used dental floss, 25% used fluoride toothpaste, and only 13.5% of them regularly visited a dentist. Less that 40% of immigrant mothers brush their children’s teeth before aged one year, 45% replaced child’s toothbrush within 3 months, and only half of the mothers regularly took their child to the dentist. Immigrant mothers had lower level of caries-related knowledge and attitudes than native mothers (*p* < .001). Compared to native group, the immigrant mothers were less likely to use of dental floss ([Adjusted odds ratio (aOR) =0.35], fluoride toothpaste (aOR = 0.29), visit a dentist in the past 2 years (aOR = 0.26), and take their children to regular dental check-up (aOR = 0.38); whereas, they were more likely to not consume sweeten beverages (aOR = 3.13).

**Conclusions:**

The level of caries-related knowledge, attitudes and oral health behaviors were found lower in immigrant mothers than native ones. The findings suggested cross-cultural caries prevention programs aimed at reducing immigrant-native disparities in child oral health care must be developed for these immigrant minorities.

## Background

Dental caries is one of the most common preventable childhood diseases, and the most common chronic disease for people worldwide. Dental caries affects general health and the quality of life in preschool children [1]. Pain caused by severe caries can cause poor chewing and affected the quantity and variety of food eaten. Also, it can make eating of high sucrose diet more likely that can compromise intake of other nutrients [1,2]. By 2011, the overall caries prevalence of Taiwanese preschoolers was 79.3%, significantly higher than in European countries and the United States, and higher than the World Health Organization (WHO) 2020 global goals for oral health: “More than 90% of preschool children free of dental caries” [3].

Since 2000, the number of immigrant women from Southeast Asia has increased rapidly in Taiwan. The majority of immigrant women are Vietnamese (64.7%) and Indonesian (20.5%), followed by Thais, Filipinos, and Cambodians. These women are colloquially called “foreign brides” or “alien brides” because their marriages were arranged by marriage brokers. The aggregate number of Southeast Asian wives in Taiwan was estimated at more than 466,000, or approximately one-third of Taiwanese marriages. One in 12 children were born of a foreign spouse by 2011 [4].

With the growing number of transnational marriages in Taiwan, health disparities have become a vital public health issue, especially in maternal and child health. Oral health inequalities exist between immigrants and non-immigrants. Previous studies conducted in Europe and the United States highlighted the worsening dental status of immigrant children [5-7]. Among immigrant children aged 4 to 6 years in Taiwan, the caries index was significant higher than in native children (6.05 vs. 3.88) [8]. The risk factors for preschool children with caries experience were found positively associated with parents in lower educational levels, scant parental attention to the child’s tooth-brushing habits, poor parental brushing habits, and higher frequency of sugar intake among parents [5,9,10], with parents playing an important role in preschool children’s caries.

Immigrant mothers have difficulty accessing the health care system because of language barriers, cultural conflicts, social and interpersonal isolation, and a lack of support systems [11]. Literature showed that unemployment, inequality and poverty is a root cause to poor health status, especially in developing countries. The social dimension of globalization encompasses security, culture and identity, inclusion or exclusion and the cohesiveness of families and communities that can impact on the health status of individual, family and society. It can also lead to deteriorating nutritional intake and inaccessibility to medical services [12,13]. Immigrant women in Taiwan were living in households with low family incomes and educations, in contrast to the native group, which has gradually led to inferior medical care for these women and their children [14]. Numerous studies reported that health insurance can increase dental care use and that is a contributing factor in the decision to seek health care services [15,16]. The Taiwanese National Health Insurance (NHI) program provides universal and comprehensive health insurance with low co-payments for dental care. Dental care insurance has 100% coverage excluding non-health problem procedures including orthodontics, prosthetic and dental implant but scaling [17]. Nevertheless, previous study [8] showed that immigrant children had lower numbers of dental restoration treatments than did native children, causing further oral health inequalities. The Taiwanese government has recently begun to pay more attention to the health care for these immigrants and provides several gratis services; however, those above services do not involve some essential oral health services. In addition, the services in the Health Care Service Project (HCSP) by the Department of Health only targets at premarital or prenatal women while the HCSP does not directly provide oral health services for preschool children, and ignores the oral health status of preschool children in new immigrant groups.

The 5-year Lay Health Advisor Approach to Promote Oral Health Program (LHA-POHP), promoting the oral health of new immigrant children, was first implemented in the Kaohsiung area in 2011. The LHA’s strategies are feasible and effective for promoting health care, especially among disparate immigrant populations [18,19]. We therefore used the baseline data of the LHA Program to explain the immigrant-native differences in caries-related knowledge, attitudes, and oral health behaviors.

## Methods

Data were obtained from the baseline data of the LHA Program. The LHA Program was originally constructed based on the PRECEDE-PROCEED framework [20] and LHA strategy to promote oral health in the children of women in transnational marriages in Taiwan. A community-based survey was conducted to collect baseline data in 2011. The self-reported questionnaire was used to assess the caries-related knowledge, attitudes, and behaviors among the immigrant and native mothers.

### Study design

The data of immigrants were collected from 20 communities selected from a list of 87 urban communities in Kaohsiung, which were selected based on the high proportion of Vietnamese and Indonesian mothers who received care from the Kaohsiung San-Min District Health Sector. The 20 communities were selected based on the availability of large number of immigrant mothers, support of village head, suitable facility for survey and oral examination.

We used a systematic random sampling method to select native children from kindergarten schools, of which 9 schools were randomly selected from a list of kindergartens located in San-Ming District provided by the Education Bureau of Kaohsiung City. All of the schools agreed to participate in the study, and all the children aged 4 to 6 years and their mothers were invited to participate.

### Participants

A sample size calculation was established by comparing proportions between the values found in native and immigrant children, using an 80% power (beta) and 5% significance (alpha) for calculation. The sample size was estimated at 422 natives and 89 immigrants. A total of 150 immigrant and 440 native mothers were administered questionnaires. The response rate for the native and immigrant mothers was 96.3% and 78.9%, respectively. The average ages of the mothers were 29.3 and 36.2 years for immigrant and native mothers, respectively.

### Instrument

#### Questionnaire development

The self-administered questionnaire was modified from an established and validated questionnaire used in a recent study [9]. The questionnaire was reviewed by a panel of experts, teachers, and the children’s mothers to assess its content and validity. To ensure that the content was understood by our participants, the questionnaire was piloted to 50 mothers of kindergarten children. Based on the results of the pilot testing, items were revised to enhance clarification and appropriateness.

The questionnaire included 63 items with close-ended response formats, such as dichotomous, ordinal, and multilevel response choices, to assess oral health behavior and its relevant variables. The questionnaire was first developed in Chinese, and then translated into Vietnamese and Indonesian by 2 bilingual experts. To ensure translation accuracy, both the Vietnamese and Indonesian versions were translated back into Chinese and then verified for accuracy by 2 senior researchers. We delivered questionnaires to all participating mothers under examination conditions overseen by trained LHA program research staff.

### Variables

#### Participant demographics

Basic demographic information consisted of maternal age, education level, employment status, and monthly household income.

#### Knowledge of the caries-related scale

The following 9-item scale statements, which we developed, were used to assess the mothers’ knowledge regarding caries and dental care services: “We have two sets of teeth in a lifetime”; “Incisors are rectangular and used for cutting food”; “Canines are triangular and used for tearing food apart”; “When newborns or young children have no teeth, we do not need to help them clean their mouths”; “Although parents kiss their children without brushing their teeth, this will not cause children to have dental caries”; “The Bureau of National Health Insurance (BNHI) provides children with fluoride varnish twice a year”; “We should pay attention to the cartoon images on a toothbrush when choosing a toothbrush”; “Dental plaque is caused by bacteria on the tooth surface”; and “The four factors of dental caries are teeth, food, bacteria, and time.” Possible responses included *true*, *false*, or *unknown* with possible scores ranging from 0 to 9; higher scores indicated a better degree of knowledge regarding caries and oral health. A 0.72 on the Kuder-Richardson reliability test was deemed acceptable.

#### Attitudinal scales

Eighteen questions regarding attitude, including attitudes toward oral hygiene (9 items), attitudes toward diet (4 items), and parental indulgence attitudes (5 items), were based the research by Skeie et al. [9]. The questionnaire was measured on a 5-point Likert scale with ratings from 1 (*strongly disagree*) to 5 (*strongly agree*). Cumulative scores were summed for each attitudinal scale, with higher scores reflecting more positive attitudes toward oral hygiene, diet, and child rearing. Cronbach’s alpha coefficients for the 3 scales ranged from 0.63 to 0.84.

#### Maternal oral health behaviors

Mothers’ oral health behaviors consisted of mother’s oral health behaviors (13 items) and their behaviors toward oral health of children (10 items). Participants responded to questions such as “How many times do you brush your teeth each day?” in the domain of the mothers’ oral health behaviors and “How old was your child the first time you assisted him or her in brushing his or her teeth?” in the domain of mothers behavior toward oral health of children. The items of the oral health behavior of mothers and their children were described elsewhere [8].

### Statistical analysis

This study explored the relationship among the variables using STATA version 10.0. The chi-square test was used to compare the new immigrant and native groups regarding the demographic distribution. A two-sample *t* test compared the new immigrant and native mothers for caries-related knowledge, attitudes toward oral hygiene, attitudes toward diet, and parental indulgence attitudes; The chi-square test was used to compare differences in the oral health behavior of both groups. A *p* value < .05 indicated a statistically significant difference between the 2 groups. In order to assess the unadjusted and adjusted association, both univariate and multivariate regression models were estimated. Only the maternal behaviors that were found to be significant associated with racial difference in the univariate regression were put into the multiple regression models. Adjusted odds ratios and 95% confidence intervals were reported for the multivariate analysis.

### Ethical considerations

This study was approved by the IRB of KMU Hospital, and we obtained letters of consent from all participants.

## Results

### The demographic distribution of immigrant and native mothers

Table 1 shows the demographic information of the native and immigrant groups. For immigrant mothers, the majority was aged under 30 years (67.61%), had no high school education (97.30%), and was unemployed (59.46%). Furthermore, 58.8% of the immigrant mothers had a household income under NTD$40,000 ($USD 1,360) per month, whereas 73.1% of native mothers had household incomes over $40,000 per month.

**Table 1:** Demographic information of immigrant and native mothers

**Variables**	**Immigrant (n = 150)**		**Native (n = 440)**	
	**N**	**%**	**N**	**%**
Age				
<30	96	67.61	36	8.65
> = 30	46	32.39	380	91.35
Educational level				
Less than high school	106	71.62	14	3.31
High school	38	25.68	153	36.17
College or higher	4	2.70	256	60.52
Occupation				
Full-time	43	29.05	239	56.10
Part-time	17	11.49	36	8.45
Unemployed	88	59.46	151	35.45
Household income				
<NTD 40,000	80	58.82	114	26.89
NTD 40,000-59,999	44	32.35	103	24.29
>NTD 59,999	12	8.82	207	48.82
Marital status				
Married, living together	142	95.95	352	81.11
Separately	4	2.70	46	10.60
Divorce or others	2	1.35	36	8.29

### Comparison of the caries-related knowledge and attitudes in immigrant and native mothers

The comparisons of average scores of caries-related knowledge and attitudes are shown in Table 2. The mean scores of knowledge, attitudes toward oral hygiene, attitudes toward diet, and parental indulgence attitudes between the immigrant and native mother groups showed significant differences (*p* < .001).

**Table 2:** Comparison of the mothers’ caries-related knowledge, attitudes toward oral hygiene, attitudes toward diet and parental indulgence attitudes in the immigrant and native group

**Variables**	**Immigrant**				**Native**				***p-value***^**a**^
	**N**	**Mean**	**SD**	**95% CI**	**N**	**Mean**	**SD**	**95% CI**	
Knowledge	137	5.15	2.08	4.80-5.51	432	7.69	1.41	7.56-7.83	<0.001
Attitudes toward oral hygiene	133	34.85	4.62	34.06-35.64	436	37.55	4.86	37.10-38.01	<0.001
Attitudes toward diet	140	15.09	2.90	14.61-15.58	435	16.28	2.81	16.01-16.54	<0.001
Parental indulgence attitudes	137	16.28	3.16	15.75-16.81	435	18.11	3.07	17.82-18.40	<0.001

^a^T-test statistics.

### Oral health behaviors of mothers and their children

Table 3 shows the distribution of the oral health behaviors of mothers and their children between the immigrant and native groups. For immigrant mothers, 68.28% reported that they replaced their toothbrush within 3 months, 13.1% replaced it in over 3 months, and 18.62% replaced when it broke. Only 37.24% of immigrant mothers reported that they flossed their teeth, compared to 74.49% of native mothers. Approximately 50% of immigrant mothers reported that they are uncertain about using fluoride toothpaste, and 62.73% of native mothers chose fluoride toothpaste.

**Table 3:** Distribution of oral health behaviors among immigrant and native mothers and their children

**Items**	**Immigrant**		**Native**		***p-value***
	**N**	**%**	**N**	**%**	
**Maternal oral health behaviors**					
***Cleaning teeth***					
How many times do you brush your teeth every day?					0.964
Once	10	6.80	27	6.18	
Twice	105	71.43	314	71.85	
Three times or more	32	21.77	96	21.97	
How many minutes do you spend brushing your teeth?					0.061
One minute	35	24.31	84	19.22	
Two minutes	53	36.81	210	48.05	
Three minutes or more	56	38.89	143	32.72	
How long do you replace your tooth brush?					<0.001
Within three months	99	68.28	325	74.54	
More than three months	19	13.10	85	19.50	
When it broke	27	18.62	26	5.96	
Do you use dental floss?					<0.001
Yes	54	37.24	327	74.49	
No	91	62.76	112	25.51	
Do you use fluoride toothpastes?					<0.001
Yes	35	24.31	276	62.73	
No	36	25.00	131	29.77	
Uncertain	73	50.69	33	7.50	
***Diet habit***					
How many times per week do you consume sweeten beverages?					0.015
Once	79	54.86	180	41.10	
Twice	20	13.89	74	16.89	
Three times or more	45	31.25	184	42.01	
How many times per week do you consume sweets?					<0.001
Once	95	65.97	200	45.98	
Twice	17	11.81	95	21.84	
Three times or more	32	22.22	140	32.18	
***Dental visit***					
Do you regularly visit a dentist?					0.010
Yes, every six months	20	13.51	103	23.41	
No	128	86.49	337	76.59	
Have you visited the dentist in the past two years?					<0.001
Yes	105	71.92	388	89.20	
No	41	28.08	435	10.80	
**Maternal behaviors toward oral health of their children**					
***Cleaning teeth of children***					
How old was your child the first time you assisted him/her in brushing his/her teeth?					<0.001
Before aged one year	55	37.67	271	61.73	
Aged one to two years	58	39.73	87	19.82	
After aged two years	33	22.60	81	18.45	
How often do you replace your child’s toothbrush?					<0.001
Within three months	94	64.38	324	73.97	
More than three months	22	15.07	95	21.69	
When it broke	30	20.55	19	4.34	
Do you use a feeding bottle before your child goes to sleep?					0.150
Yes	46	31.51	111	25.40	
No	100	68.49	326	74.60	
Do you direct your child to brush his/her teeth?					0.739
Yes	145	97.97	427	97.49	
No	3	2.03	11	2.51	
How many minutes does your child spend brushing his/her teeth?					<0.001
One minute	53	40.15	189	47.13	
Two minutes	39	29.55	165	41.15	
Three minutes or more	40	30.30	47	11.72	
***Diet habit of children***					
How many times per week do your children consume sweets?					0.013
Once	41	27.89	77	17.78	
Twice	26	17.69	112	25.87	
Three times or more	80	54.42	244	56.35	
How many times per week do your children consume sweeten beverages?					0.054
Once	62	42.76	145	33.03	
Twice	30	20.69	128	29.16	
Three times or more	53	36.55	166	37.81	
***Dental visit of children***					
Do you regularly take your child to the dentist?					<0.001
Yes, every six months	80	53.69	379	86.53	
No	69	46.31	59	13.74	
Have you taken your child to the dentist in the past two years?					<0.001
Yes	104	71.72	378	86.50	
No	41	28.28	59	13.50	

The following 2 questions regarding maternal diet behaviors were significantly different for the 2 groups: “How many times per week do you consume sweeten beverages?” 31.25% of immigrant stated 3 or more times per week compared to 42.01% in native mothers; for “How many times per week do you consume sweets?” 22.22% of immigrant mothers responded to 3 times or more per week; however, 32.18% of native mothers answered 3 times or more per week. For mother’s dental visit behaviors, both groups did not visit the dentist regularly, with rates of 86.49% and 76.59% in immigrant and native mothers, respectively (*p* = .010). Fewer immigrant mothers (71.92%) visited a dentist in the past 2 year, compared to native mothers (89.20%) (*p* = .001).

The 3 following questions regarding maternal behaviors toward cleaning teeth of children showed significant immigrant-native differences: “How old was your child the first time you assisted him/her in brushing his/her teeth?”; 62.33% of immigrant mothers responded with 1 year of age or older, but 61.73% of native mothers responded with under 1 year of age; for “How often do you replace your child’s toothbrush?” 64.38% of immigrant mothers stated within 3 months, but 73.97% of native mothers replaced it within 3 months; only 30.30% of immigrant mother responded with 3 minutes or more to “How many minutes does your child spend brushing his/her teeth?”

Regarding dietary habits of children, 54.42% of immigrant mothers reported 3 times or more a week to “How many times per week do your children consume sweets?” compared to 56.35% of native mothers (*p* = .013). For dental visit of children, only 53.69% of immigrant mothers brought their children for regular dental check-ups, but 86.53% of native mothers brought theirs. Fewer immigrant mothers (71.72%) brought their children to visit a dentist in the past 2 years, compared to native mothers (86.50%). Both questions were significantly different, as shown in Table 3 (*p* < .001).

### Racial difference related to oral health behaviors

Table 4 and Table 5 shows multivariate logistic regression analysis of racial difference related to maternal oral health behaviors and maternal behaviors toward oral health of their children. After adjusting for potential covariates, the immigrant mothers were less likely to use of dental floss [Adjusted odds ratio (aOR) = 0.35; 95% CI = 0.17-0.75], use of fluoride toothpaste (aOR = 0.29; 95% CI = 0.13-0.64) and dental visit in the past 2 years (aOR = 0.26; 95% CI = 0.10-0.65); whereas they were more likely to not consuming sweeten beverages (aOR = 3.13; 95% CI = 1.47-6.67). Mothers who have higher level of caries-related knowledge were more likely to use of dental floss (aOR = 1.14; 95% CI = 1.00-1.31), fluoride toothpaste (aOR = 1.18; 95% CI = 1.03-1.34) and dental visit in the past 2 years (aOR = 1.19; 95% CI = 1.02-1.40). Mothers who have positive attitude toward oral health were more likely to have regular dental check-ups every 6 months (aOR = 1.03; 95% CI = 1.01-1.06).

**Table 4:** Multivariate logistic regression analysis of racial difference related to maternal oral health behaviors

**Characteristics**	**Model A**		**Model B**		**Model C**		**Model D**		**Model E**	
	**aOR**	**(95% CI)**	**aOR**	**(95% CI)**	**aOR**	**(95% CI)**	**aOR**	**(95% CI)**	**aOR**	**(95% CI)**
**Race**										
Native	1		1		1		1		1	
Immigrant	0.35	(0.17-0.75)	0.29	(0.13-0.64)	3.13	(1.47-6.67)	1.24	(0.42-3.69)	0.26	(0.10-0.65)
**Age**										
> = 30	1		1		1		1		1	
<30	1.08	(0.60-1.95)	0.66	(0.40-1.19)	0.45	(0.26-0.81)	0.88	(0.41-1.88)	2.34	(1.08-5.04)
**Educational level**										
College or higher	1		1		1		1		1	
High school	1.23	(0.73-2.08)	0.85	(0.53-1.36)	1.20	(0.76-1.88)	0.38	(0.21-0.71)	0.96	(0.47-1.99)
Less than high school	0.67	(0.29-2.08)	0.97	(0.40-2.35)	0.85	(0.37-1.92)	0.70	(0.22-2.21)	1.18	(0.39-3.55)
**Household income**										
>NTD 59,999	1		1		1		1		1	
NTD 40,000-59,999	1.52	(0.87-2.67)	1.33	(0.80-2.23)	1.07	(0.66-1.73)	0.87	(0.49-1.53)	1.04	(0.49-2.19)
<NTD 40,000	0.89	(0.52-1.50)	1.23	(0.74-2.05)	1.09	(0.67-1.76)	0.69	(0.37-1.30)	1.03	(0.50-2.13)
**Knowledge**	1.14	(1.00-1.31)	1.18	(1.03-1.34)	0.97	(0.86-1.10)	1.18	(0.98-1.42)	1.19	(1.02-1.40)
**Attitudes**	0.98	(0.96-1.01)	1.02	(0.99-1.05)	1.01	(0.99-1.03)	1.03	(1.00-1.06)	1.00	(0.97-1.04)

aOR was adjusted for covariates in the table.

Model A: Maternal dental floss use (Yes vs. No).

Model B: Maternal fluoride toothpastes use (Yes vs. No).

Model C: Maternal consumption of sweeten beverages per week (Once or less vs. Twice or more).

Model D: Maternal regular dental check-up (Yes vs. No).

Model E: Visited a dentist in the past two years (Yes vs. No).

**Table 5:** Multivariate logistic regression analysis of racial difference related to maternal behaviors toward oral health of their children

**Characteristics**	**Model F**		**Model G**		**Model H**		**Model I**	
	**aOR**	**(95% CI)**	**aOR**	**(95% CI)**	**aOR**	**(95% CI)**	**aOR**	**(95% CI)**
**Race**								
Native	1		1		1		1	
Immigrant	0.76	(0.37-1.59)	1.12	(0.52-2.41)	0.38	(0.17-0.83)	0.73	(0.31-1.75)
**Age**								
> = 30	1		1		1		1	
<30	1.00	(0.58-1.74)	0.60	(0.34-1.06)	0.66	(0.36-1.23)	0.81	(0.42-1.56)
**Educational level**								
College or higher	1		1		1		1	
High school	0.85	(0.53-1.35)	1.29	(0.76-2.16)	0.55	(0.29-1.04)	0.62	(0.32-1.20)
Less than high school	1.00	(0.44-2.28)	1.13	(0.47-2.69)	0.80	(0.30-2.13)	0.10	(0.15-1.10)
**Household income**								
>NTD 59,999	1		1		1		1	
NTD 40,000-59,999	1.26	(0.76-2.09)	0.74	(0.42-1.28)	0.64	(0.32-1.27)	0.58	(0.29-1.12)
<NTD 40,000	0.88	(0.54-1.43)	0.63	(0.37-1.07)	0.81	(0.42-1.56)	0.92	(0.46-1.84)
**Knowledge**	1.23	(1.08-1.40)	1.05	(0.92-1.19)	1.11	(0.97-1.28)	0.97	(0.84-1.13)
**Attitudes**	1.02	(0.99-1.04)	1.04	(1.01-1.07)	1.05	(1.02-1.08)	1.03	(0.99-1.06)

aOR was adjusted for covariates in the table.

Model F: The first time mother assisted child in tooth brushing (before aged one year vs. after aged one year)

Model G: Replace child’s toothbrush (Within three months vs. More than three months/broke).

Model H: Child regular dental check-up (Yes vs. No).

Model I: Visited a dentist in the past two years (Yes vs. No).

Compare to native mothers, the immigrant mothers were less likely to take their children to regular dental check-up (aOR = 0.38; 95% CI = 0.17-0.83). Those mothers who have high level of knowledge score were more likely to assist child in brushing before the age of one (aOR = 1.23; 95% CI = 1.08-1.40). Mothers who have positive attitudes toward oral health were more likely to replace child’s toothbrush within three months (aOR = 1.04; 95% CI = 1.01-1.07) and take child regular dental check-ups every 6 months (aOR = 1.05; 95% CI = 1.02-1.08).

## Discussion

Our study is the first to identify a correlation between immigrant-native disparities and oral health behaviors in urban mothers and their children in Taiwan. According to our results, immigrant-native differences in knowledge, attitude and behaviors toward oral health of mothers and their children were observed. Our findings also confirmed that racial difference was related to oral health behavior of mothers and their children.

The findings of this study showed significantly lower level of caries-related knowledge in immigrant mothers which indicate a higher demand for immigrant mothers for oral health knowledge. Moreover, our study shows that immigrant mothers have more negative attitudes toward oral hygiene, diet, and parental indulgence compared to native mothers. Attitudes are derived from targeted beliefs; each belief and behavior are connected to a specific result, leading to the implementation of production [21].

In this study, the frequency of immigrant mothers’ oral health behaviors was lower than for native mothers. Less than 40% of immigrant mothers assisted their children in tooth brushing before the age of one. Skeie [9] found that the rate of helping children younger than 1 year brush their teeth for immigrant mothers was lower than for native mothers. Fewer regular dental checkups in immigrant mothers and their children were also observed in our study. Cultural differences in dental attendance and self-care practices of children and their parents, such as higher percentage of sweet consumption and lower percentage of dental health practice among immigrant children have been reported in previous studies [22,23]. The study showed that at age seven, 53% of native Danish and 84% of Albanian children were founded infected with dental caries. The mean caries infection was more serious among Albanian children than native Danish children (13.8 vs. 3.5). Socio-behavioral factors are responsible in making dental caries prevalence and severity among these two groups of children. Despite dental visits for children aged 6 years and under being free of charge under Taiwanese health insurance coverage, our study found that few immigrant mothers were aware that “The Bureau of National Health Insurance provides children with fluoride varnish twice a year”, indicating that mothers lack of health information regarding this protective service.

A greater level of knowledge and a more positive attitude toward oral hygiene are a prerequisite for successful caries preventive behaviors. Studies have reported that there is a link between the behavior of preschool children, such as brushing habits, and mothers’ awareness, attitudes and behaviors [9,10]. In the present study, mothers who have higher level of caries-related knowledge were more likely to use of dental floss, fluoride toothpaste and dental visit in the past 2 years, as well as assist child in tooth brushing before the age of one. Our findings also showed that positive attitude toward oral health was associated with the oral hygiene behavior. Mothers who have positive attitudes toward oral health were more likely to replace child’s toothbrush within three months and take their child regular dental check-ups.

A positive relationship was found between preventive health care for children and the SES of their families [24]. Dental caries can be prevented by professionally applied topical fluorides, dental sealants, and the use of fluoride toothpastes. However, children from low income families were found to have worse oral health outcomes, fewer dental visits, and fewer protective sealants [25]. Immigrant mothers are often limited by their language barriers and low SES, resulting in a lower utilization rate of preventive health care and services. In addition, a lack of message readiness may explain the lower access to dental visits. Studies have reported that a link exists between access to health information and socioeconomic status [26,27]. In this study, immigrant families showed evidence of a low economic and living standard; more than half were living in households with the lowest household income and parental education levels. Exposure to media messages has increased in Taiwanese society, but health information is still not readily available for lower socioeconomic segments of the population.

The data in our study was collected from self-reported questionnaires. Mothers may not have presented the actual situation due to social desirability considerations. Nevertheless, the questionnaires were anonymously recorded; consequently, the validity doubts regarding answer errors should have been avoided. The data obtained in this study may not be generalizable to all communities within our county. However, the study methodology and research network might be extended to areas where Southeast Asian immigrant women in arranged transnational marriages are common.

## Conclusions

Immigrant-native differences regarding caries-related knowledge, attitudes, and behaviors were identified. A lower level of knowledge, negative attitudes toward oral hygiene, and the frequency of oral health behavior in immigrant mothers may affect the oral health of their children. A lack of message readiness among these immigrant mothers with a language barrier may explain the lower access to dental visits. The findings suggested a need of designing effective health communication in cross-cultural caries prevention programs for these immigrant minorities to raise risk awareness and dental services aimed at reducing immigrant-native disparities in child oral health care.

## Competing interests

The authors declare that they have no competing interests.

## Authors’ contributions

CCC is the primary writer of the manuscript and participated in the study implementing. SJC and CCT conceived of the study, and had made substantial contributions to conception and design, and revised the manuscript critically for important intellectual content. YCL, CCH and FLC participated in the design of the study, acquisition of data, and revised the manuscript. CHL assisted in statistical analysis, interpretation of data and draft the statistical analysis of manuscript. TC, CSC and YYL revised the manuscript critically for important intellectual content. HLH is the principal investigator of this project. She contributed to the design, sampling protocol and method development, statistical analysis and interpretation of the results, and has been involved in drafting and revising the manuscript. All authors read and approved the final manuscript.

## Pre-publication history

The pre-publication history for this paper can be accessed here:

http://www.biomedcentral.com/1472-6831/14/3/prepub
